# Development of slow oscillation–spindle coupling from infancy to toddlerhood

**DOI:** 10.1093/sleepadvances/zpae084

**Published:** 2024-11-16

**Authors:** Eva-Maria Kurz, Lisa Bastian, Matthias Mölle, Jan Born, Manuela Friedrich

**Affiliations:** Institute of Medical Psychology and Behavioral Neurobiology, University of Tübingen, Tübingen, Germany; Department of Child and Adolescent Psychiatry, Psychosomatics and Psychotherapy, University Hospital of Psychiatry and Psychotherapy, University of Tübingen, Tübingen, Germany; Institute of Medical Psychology and Behavioral Neurobiology, University of Tübingen, Tübingen, Germany; Max Planck School of Cognition, Max Planck Institute for Human Cognitive and Brain Sciences, Leipzig, Germany; Center of Brain, Behavior and Metabolism (CBBM), University of Lübeck, Lübeck, Germany; Institute of Medical Psychology and Behavioral Neurobiology, University of Tübingen, Tübingen, Germany; Center for Integrative Neuroscience, University of Tübingen, Tübingen, Germany; German Center for Mental Health (DZPG), site Tübingen, Germany; Department of Psychology, Humboldt-University, Berlin, Germany; Department of Neuropsychology, Max Planck Institute for Human Cognitive and Brain Sciences, Leipzig, Germany

**Keywords:** development, infancy, slow oscillation–spindle coupling, spindle, slow oscillation

## Abstract

Sleep has been demonstrated to support memory formation from early life on. The precise temporal coupling of slow oscillations (SOs) with spindles has been suggested as a mechanism facilitating this consolidation process in thalamocortical networks. Here, we investigated the development of sleep spindles and SOs and their coordinate interplay by comparing frontal, central, and parietal electroencephalogram recordings during a nap between infants aged 2–3 months (*n* = 31) and toddlers aged 14–17 months (*n* = 49). Spindles and SOs showed quite different maturational patterns between age groups, as to topography, amplitude, and density. Notably, spindle–SO co-occurrence in the infants did not exceed chance levels and was increased to significant levels only in the toddlers. In the infants, the slow SO upstate over frontocortical regions was even associated with a significant decrease in spindles, contrasting with the adult-like increase in spindles seen in toddlers. These results point to an immature processing in thalamocortical networks during sleep in early infancy, possibly diminishing the efficacy of sleep-dependent memory formation at this age.

**Statement of Significance**

Sleep is thought to support memory consolidation from early life on. However, the underlying mechanisms may be different. The precise nesting of sleep spindles into slow oscillation (SO) upstates has been identified in older children and adults as a key mechanism supporting memory consolidation during sleep. Here, we analyzed the precision of this SO–spindle coupling in 2–3 months old infants and 14–17 months old toddlers. Although the density of spindles and SOs was higher in the infants than toddlers, only the toddlers but not the infants exhibited the characteristic coupling of spindles into SO upstates. This novel finding suggests that thalamocortical networks at 3 months of age might still be too immature for efficient processing and consolidation of information during sleep.

Among the myriads of physiological phenomena during sleep, the interplay between sleep spindles (9–16 Hz) and slow oscillations (SOs; 0.5–1 Hz) during non-rapid eye movement (non-REM) sleep constitutes a hallmark of mammalian sleep. SOs originate from the neocortex as alternations between hyperpolarization and depolarization of large groups of cortical neurons [1, 2]. Governed by the depolarized SO upstate, neurons of the reticular thalamic nucleus, in turn, generate sleep spindles [3] that propagate to cortical regions. Here, spindles gate dendritic calcium shifts to promote synaptic plasticity mediating memory consolidation that is relevant for future perceptual and cognitive processing [4–7]. Thus, the coalescence between spindles and SOs provides a powerful neurophysiological biomarker for cognitive functioning.

Both SOs and spindles exhibit developmental changes, yet these changes follow distinct trajectories. During early childhood (2–8 years), SOs are most prominent and have their origin in posterior brain regions, starting to gradually shift toward a mature predominance in anterior areas at around 8–11 years of age [8]. Conversely, spindles show already within the first year of life a topographical shift from frontopolar to central regions, thereafter, shifting again toward anterior regions during childhood [9]. Across adolescence, spindles become more prominent over centro-parietal areas. In addition to these topographical changes, both spindles and SOs undergo distinct and dynamic alterations in density, amplitude, and frequency [10, 11], which appear to be particularly pronounced for spindle density: Spindle density increases linearly from birth to ~4 months, followed by a slight decrease from 4 to 10 months, and remaining then at relatively stable levels between 1 and 3 years [9]. The oscillatory spindle frequency peaks in the first year of life (13.1 Hz), sharply decreasing thereafter (from 1 to 2 years) to around 11.2 Hz [9]. The adult-like divergence into frontocortical slow and centro-parietal fast spindles, respectively, begins to develop at ~6 months [9]. While SO density does not appear to change in the first 9 months of life, their amplitude and slope increase [12]. Given these seemingly independent developmental trajectories of spindles and SOs, the development of their interplay and coalescence is specifically interesting.

Indeed, SO–spindle coupling has recently gathered significant attention, as it is believed to be a critical mechanism for sleep-dependent memory consolidation across development [6, 13–15]. Together with hippocampal sharp wave-ripples (80–120 Hz), the precise coordination of spindles and SOs sets a temporal framework for a hippocampal-cortical dialogue underlying systems memory consolidation [5, 16, 17]. There is evidence indicating that the temporal coordination of SOs and spindles improves across childhood and adolescence [11, 15], which might be specifically driven by the emergence of faster, adult-like spindle frequencies [10]. However, not much is known about how SOs and spindles are coordinated before the age of 2 years. One study reported the presence of a coalescence between spindles and SOs in 6-month-old infants [18]. Interestingly, none of the assessed SO and spindle parameters (including SO–spindle co-occurrence) was correlated with the current behavioral developmental state of the infants, but fast spindle density at 6 months predicted development at 12 months.

Here, we aimed to investigate the development of SO–spindle coupling during non-REM sleep in infants (2–3 months) and toddlers (14–17 months). Considering the evidence of significant SO–spindle co-occurrence in 6-month-olds [18], we investigated whether such coupling could occur even earlier, i.e. at 2–3 months of age. We compared measurements in these infants with those in toddlers, aged 14–17 months, based on evidence indicating that spindle characteristics undergo the most prominent changes during this period from infancy to toddlerhood. We hypothesized that any coupling of spindles to SOs would be less pronounced in infants compared to toddlers.

## Materials and Methods

### Participants

We analyzed sleep EEG recordings from a nap study comprising 87 participants. Six participants were excluded due to less than 10 minutes of artifact-free non-REM sleep. One recording was excluded due to recording failure. The final sample comprised 31 infants (age: M = 2.65 months, SEM = 0.09, range = 2–3, 12 male and 19 female) and 49 toddlers (age: M = 15.2 months, SEM = 0.15, range = 14–17, 29 male and 20 female). Of these, five participants were tested two times and are thus part of both the 2–3 months age group and the 14–17 months age group. Birthweight ranged from 2750 to 4580 g and did not differ between infants (M = 3544, SEM = 90.78) and toddlers (M = 3648, SEM = 65.42, *t*(59.26) = −0.93, *p* = .357). Three participants from the infant age group and one participant from the toddler age group had an APGAR score of 8 after 10 minutes. All others had scores of 9 and 10 with a median of 10 in both age groups.

Participants were recruited in the area of Berlin, Germany. All parents gave written informed consent and the ethics committee of the Department of Psychology of the Humboldt University of Berlin approved the study. All participants were born at term and no developmental delay and no major sleep problems (as indicated in a 1-week sleep diary, see below) were reported.

### Procedure

All infants and toddlers were part of a larger study investigating sleep’s effect on the consolidation of syntactic regularities, which were extracted during a familiarization period while listening to an unknown language. Briefly, infants and toddlers participated in two sessions (encoding/familiarization and test session) with a retention interval of 0.5–2 hours in between. Participants slept on average 58 minutes (SEM = 2.54) during this period. For a similar design see Friedrich et al. [19]. For the current question of interest, only sleep EEG recordings were analyzed.

Prior to the experimental session, parents filled out a one-week sleep diary, in which they indicated their child’s sleep and wake times during the day- and nighttime. Additionally, they indicated whether their child had any sleep problems during that time. Diary entries are available from 28 infants and 47 toddlers. We provide average total sleep time (TST) during the day and night (in hh:mm) as well as the number of naps during the day and the number of awakenings during the night in Supplementary Table S1.

### Sleep recordings

Sleep was continuously recorded with a sampling rate of 256 Hz with a portable PSG device (SOMNOscreen 10-20, SOMNOmedics GmbH, Randersacker). EEG was recorded from F3, Fz, F4, C3, C4, P3, Pz, and P4, as well as the left and right mastoids. Cz was used as a recording reference and Fpz as ground. Vertical and horizontal electrooculogram and electromyogram were recorded bipolarly from electrodes placed right and left of the eyes, above and below the right eye and under the chin, respectively. For sleep EEG scoring, based on standard criteria [20, 21], the EEG was offline re-referenced to the average of the mastoids and filtered between 0.3 and 35 Hz. Sleep scoring was done by an experienced scorer using the software REMBRANDT 9 (Natus Medical, Pleasanton, USA). For the purpose of the present study, we obtained TST, and time spent in N2 and N3 sleep. Artifactual epochs were marked during the scoring process and excluded from further analyses.

The timing of the sleep onset of the naps (any stage of sleep) was based on the sleep scoring except for two participants, where the parent report of sleep onset was used due to the following reasons: one participant fell asleep before the start of the recording and for another participant, scoring was only possible ~40 minutes after recording start due to technical issues. For a subsample (*n* = 28 infants, *n* = 37 toddlers) we provide the wake time (in minutes) since the last nap, calculated as the difference between sleep onset and the end of the preceding nap (parent report) in Supplementary Table S2.

### Spindle detection

For sleep spindle detection during non-REM sleep (N2 and N3 sleep), we first identified frequency peaks (between 9 and 17 Hz) in the power spectrum. Power spectra were computed for each participant (MATLAB’s *pwelch* function, 5-second windows, 50% overlap) for the average of frontal (F3, Fz, and F4), central (C3, Cz, and C4) and parietal (P3, Pz, and P4) channels. In MATLAB, power spectra were then subjected to the Python-based toolbox “fitting oscillations & one over f” (FOOOF, [22]) for the calculation of the aperiodic signal (1/*f*-like component). In brief, the power spectrum was modeled as the sum of aperiodic and periodic activity. After an initial fit of the aperiodic activity, which was subtracted from the original power spectrum, peaks were iteratively fitted by Gaussian functions. This successive fitting and subtraction procedure was repeated as long as a threshold (see below) was exceeded, followed by a final re-fit of the aperiodic activity. Models were fitted across the frequency range of 0.5–25 Hz. Threshold configurations included the fit of a maximum of 3 peaks with a bandwidth between 0.5 and 12 Hz. The minimum peak height was 0, although it had to exceed 2 SDs from the residual signal. Model fit was inspected based on guidelines provided in Ostlund et al. [23]. The mean model fit (*R*²) was 0.996 (SD = 0.002, range: 0.989–0.999) and the average error was 0.055 (SD = 0.015, range: 0.027–0.112). The identified frequency peaks were considered as center frequencies for spindle detection. For each participant, the fit of the peaks was visually inspected after the subtraction of the aperiodic signal from the power spectra. In case a peak was not fit appropriately, the peak was determined manually. If a participant did not show a clear peak in one of the channels, the average peak frequency of the other channels of this individual was used. A minority of participants (on average ~9% of the infants, ~13% of the toddlers) showed two frequency peaks within the spindle range. For further analyses, only peaks above 12 Hz were considered (infants: M = 13.6 Hz, SD = 0.71, toddlers: M = 14.1 Hz, SD = 0.82).

Spindle detection was done using the MATLAB-based open-source toolbox SleepTrip (https://sleeptrip.org/; RRID:SCR_017318) in MATLAB 2023b, which is based on Mölle et al. [24] and has previously been used in adult and developmental studies [11, 13]. To ensure validity, the detected events are visually inspected in the continuous EEG for random participants.

For each individual, the re-referenced, unfiltered EEG signal was bandpass filtered (two-pass Butterworth filter, filter order 4) ±2 Hz around the identified frequency peaks. Then, for each sample point, the root mean square (RMS) in a 0.2-second time window is calculated, which is further smoothed by averaging time windows of 0.2 seconds. If the duration of the smoothed moving RMS lies between 0.5 and 3 seconds and exceeds the filtered signal in the respective channel by 1.5 SDs, a spindle is detected. For each participant and each channel, we determined the density of spindles (number per minute) and the amplitude (maximum peak to trough potential), averaged across frontal, central, and parietal channels.

### SO detection

Detection of SOs during non-REM sleep (N2 and N3 sleep) was similarly done using the toolbox SleepTrip, which is based on Mölle et al. [24] and has previously been used in adult and developmental studies [11, 13]. Also here, detected events of random participants were inspected in the continuous EEG to check the validity of the algorithm.

The signal was first high-pass filtered at 0.3 Hz (two-pass Butterworth, fourth order) and then low-pass filtered at 4 Hz (two-pass Butterworth, sixth order). In the filtered signal, all intervals with consecutive positive-to-negative zero crossings were marked as a potential SO. An SO was detected when the following criteria were met: frequency between 0.5 and 1.25 Hz (corresponding to a duration of 0.8–2 seconds), amplitude 1.25 times greater than the mean amplitude of the other potential SOs within the channel, and down-peak (trough) potential 1.25 times lower than the mean down-peak potential of the other SOs in this channel. For each participant and channel, we calculated the density of SOs (number per minute), the amplitude (maximum trough to peak potential), the ascending slope (ratio between the trough potential and the time delay to the following up-zero crossing; cf. [25]) and duration (time between the two positive-to-negative zero crossings), which were then averaged across frontal, central, and parietal channels.

### Co-occurrence of SOs and spindles

In the first step, we looked for each detected spindle, and whether its center (i.e. the maximum trough) occurred within the two positive-to-negative zero crossings of an SO. The ratio of spindles co-occurring with an SO was further averaged across frontal, central, and parietal channels.

In the second step, the ±1000 ms around an SO trough were subdivided into 20 100 ms bins to calculate peri-event time histograms (PETHs; cf. [26, 27]). For each bin, occurrences of spindle centers (i.e. the maximum trough) were summed across all SOs of a channel of an individual and then normalized by the number of spindles co-occurring with SOs in the respective channel. The resulting occurrence probabilities were averaged across frontal, central, and parietal channels. To analyze whether occurrence probabilities were clustered toward a specific time window of the SOs, the bins within each participant and channel were shuffled 1000 times and averaged to create surrogate data for statistical testing.

In the third step, we looked at the time-frequency representations (TFRs) of SOs using the open-source toolbox FieldTrip [28]. For this, the ±3 seconds around the SO trough were extracted and subjected to time-frequency analyses using Morlet wavelets with linearly increasing cycles from 4 to 12, in a frequency range from 5 to 20 Hz in steps of 0.5 Hz and 3.9 ms. The power in each channel was normalized by the average power in the ±1.5 seconds around the SO trough, represented as percentage change, with positive values indicating an increase in power from the baseline. Normalized power was then averaged across frontal, central, and parietal channels in each individual.

Lastly, we investigated the phase-amplitude coupling of SOs and spindles (cf. [16, 29]). For this, the signal was filtered in the low- and high-frequency range (same ranges and filters as for the detection of SOs and spindles, respectively). Using the Hilbert transform, the instantaneous phase and amplitude were calculated for the low-frequency range and high-frequency range time series, respectively. For each spindle that coincided with an SO in the two positive-to-negative zero crossings of an SO, the SO phase at the maximum spindle amplitude was extracted. To calculate representative circular averages and vector lengths, only channels with at least five co-occurring events were included in the following analyses. Using the circStat toolbox [30] in MATLAB, we calculated for each participant and each channel the mean resultant vector length (*circ_r*) and mean preferred phase (*circ_mean*) of coupling. The mean resultant vector length, as a measure of how consistently maximum spindle amplitudes are clustered toward the same SO phase, ranges from 0 to 1, with 1 indicating maximum clustering toward the same phase. The mean preferred phase of coupling describes for an individual the average SO phase at which spindles show their maximum amplitude, with 0° representing the SO upstate and ±180° representing the SO downstate. The same measures were calculated across participants’ mean preferred phase of coupling within an age group.

### Statistical analyses

Statistical analyses were performed in R version 4.3.2 (R Core Team, [31]) and MATLAB 2023b. Characteristics of spindles and SOs were analyzed with linear mixed-effects models using the R package *lme4* [32]. We included fixed effects for age group (infants vs. toddlers) and channel (frontal vs. central vs. parietal), as well as random intercepts for participants. We used the R package *lmerTest* [33] to obtain F-statistics and *p*-values based on the Type III sum of squares and Satterthwaite’s method. Any posthoc pairwise comparisons were Bonferroni-corrected and calculated using the *emmeans* package [34]. The model for co-occurrence rates was extended by the fixed effect type (observed vs. chance). Observed co-occurrence rates are the percentage of spindles co-occurring with an SO, while chance was calculated for each individual and channel as the percentage of non-REM sleep with SOs (total SO duration/non-REM sleep duration × 100). If spindles are distributed equally across non-REM sleep and do not tend to occur together with an SO, their proportion of co-occurring with an SO should not significantly deviate from the proportion of non-REM sleep with SOs.

Statistical analysis of the PETHs against surrogate data was done in MATLAB using the FieldTrip toolbox [28]. Separately for each channel average and age group, we calculated dependent samples *t*-tests, which were corrected for multiple comparisons using cluster-based permutation tests [35], with 5000 permutations and a cluster alpha-level of 0.05 (two-tailed), Monte Carlo Method. Similarly, baseline-normalized TFRs were tested against zero and likewise corrected for multiple comparisons using cluster-based permutation tests. In a second step, we compared the baseline-normalized TFRs between age groups using independent samples *t*-tests, again corrected for multiple comparisons using cluster-based permutation tests.

For statistical analyses of phase-amplitude coupling, we first used Rayleigh tests to calculate whether preferred phases in each channel of each individual are nonuniformly distributed. A significant test would indicate a nonuniform distribution of maximum spindle amplitudes across the SO cycle. Analysis of the mean resultant vector length was similarly done using a linear mixed-effects model. Group differences in the mean preferred phase of coupling were analyzed using the Watson–Wheeler test in R using the package *circular* [36], which is a nonparametric alternative for the Watson–Williams test. The Watson–Williams test was not applicable due to a too low average resultant vector length (<0.45).

## Results

### Non-REM sleep characteristics

For both infants and toddlers, the sleep onset of the naps was spread across the daytime and did not differ between the age groups (*p* = .502). However, toddlers spent longer times awake prior to the experimental nap compared to infants (*p* < .001). Means (and standard errors) for the start of the naps, wake time prior to the nap, and time spent in non-REM sleep are reported in Supplementary Table S2. Briefly, the 2- to 3-month-old infants took shorter naps in the retention period than the 14- to 17-month-old toddlers (*p* = .009). While the two groups did not differ regarding the amount of time spent in N2 sleep, the infants exhibited less N3 sleep (min: *p* < .001, % of TST: *p* = .003). Controlling for the participants’ sex showed that female participants slept longer (*p* = .017) and had longer N2 sleep (*p* = .022) than male participants. Male and female participants did not differ in their proportion of N2 sleep or in the duration and proportion of N3 sleep (all *p* > .116). All subsequent analyses were done twice—with and without controlling for sex. Since none of the results changed when including sex as a covariate and the covariate was not significant (all *p* > .141), we will only report the results of the models that did not control for sex.

### Spindle characteristics

**Figure 1.**
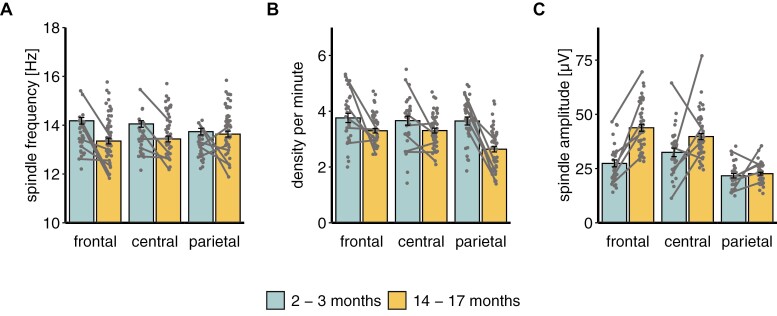
Sleep spindle characteristics dependent on age group and channel. Blue (left) bars depict infants (2–3 months) and yellow (right) bars toddlers (14–17 months), separately for frontal, central, and parietal channels. Gray lines depict the participants for whom recordings were available at both ages. Please note, estimated marginal means are depicted in (A) (see also Supplementary Table S3).

Spindle frequency yielded a significant interaction of age group × channel (*F*(2,160) = 24.07, *p* < .001). Posthoc pairwise comparisons showed a higher spindle frequency in infants than toddlers in frontal (*t*(231) = 6.16, *p* < .001) and central channels (*t*(231) = 4.57, *p* = .001), but not in parietal channels (*t*(231) = 0.75, *p* > .999; Figure 1, A). In infants, parietal channels had the smallest spindle frequency compared to frontal (*t*(171) = 4.46, *p* < .001) and central channels (*t*(171) = 3.17, *p* = .016), with no difference between frontal and central channels (*t*(171) = 1.29, *p* > .999). Within toddlers, spindle frequency was higher at parietal than frontal channels (*t*(171) = −3.58, *p* = .004), with no further channel differences (all *p* > .123; Figure 1, A; Supplementary Table S2). Controlling for the sleeping patterns of the participants by adding the average number of naps per day as a covariate, revealed a significant effect of the covariate (*F*(1,220.08) = 30.39, *p* < .001, *r* = −0.21). The abovementioned results stayed the same, except that additionally infants had higher spindle frequencies than toddlers also in parietal channels (*t*(231) = 5.85, *p* < .001).

For spindle density and amplitude, we each found significant effects of channel (all *p* <.001), age group (all *p* <.001) as well as an interaction between age group and channel (see Supplementary Table S3 for means and SEs). The significant interaction (*F*(2,158.39) = 11.71, *p* <.001) for spindle density showed a higher spindle density in infants than toddlers in all three channels (all *p* <.001; Figure 1, B). There was no difference in density between the three channels in the infants (all *p* >.999). Toddlers showed the smallest density in the parietal as compared to frontal (*p* <.001) and central (*p* <.001) channels, with no difference between frontal and central channels (*p* >.999). The interaction of age group and channel (*F*(2,165.33) = 27.81, *p* <.001) for spindle amplitude did not show any difference between infants and toddlers for the parietal channel (*t*(246) = −1.37, *p* >.999). However, infants had smaller amplitudes at frontal (*t*(246) = −9.74, *p* <.001) and central channels than toddlers (*t*(246) = −4.79, *p* <.001). In both age groups, spindles in parietal channels had the smallest amplitudes compared to frontal and central channels (all *p* <.007). While amplitudes were greater in central than frontal channels (*t*(170) = −3.08, *p* =.022) in infants, the reverse effect was observed in toddlers (*t*(170) = 3.08, *p* =.022; Figure 1, C). Exploring, whether spindle density and amplitude might be influenced by the participants’ sleeping patterns did not show a significant effect of the average number of naps per day (*p* = .521 and *p* = .473, respectively) and no change in the above-reported results. Our results similarly stayed unchanged when exploring possible effects of the timing of the experimental nap (*p* = .010) and the waketime since the last nap (*p* = .438) on spindle amplitude, although the timing per se was significant.

### SO characteristics

**Figure 2.**
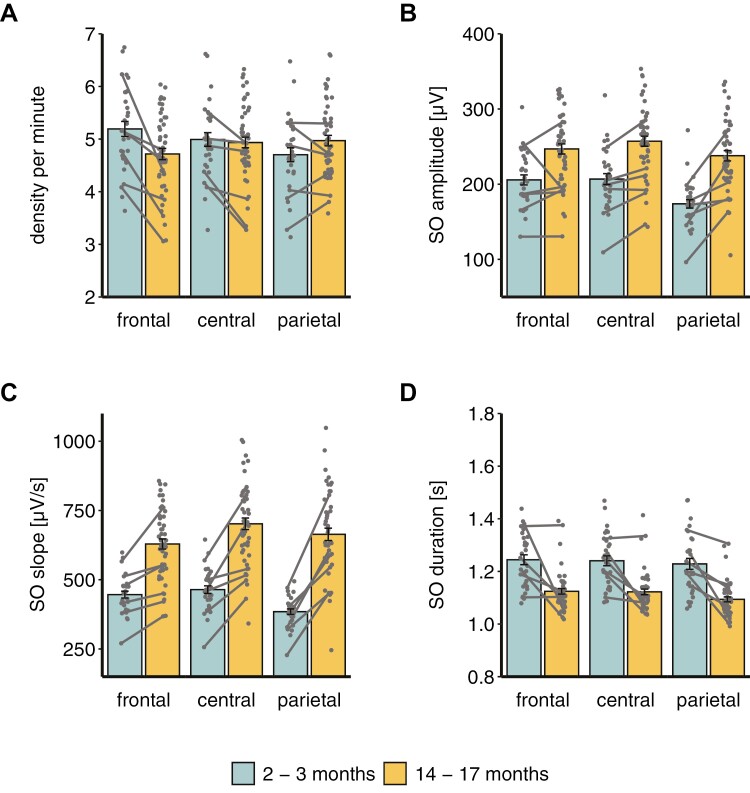
Slow oscillation characteristics dependent on age group and channel. Gray lines depict the participants for whom recordings were available at both ages.

SO density, amplitude and slope each showed significant main effects of age group (all *p* < .007) and channel (all *p* < .001), as well as significant interactions between these factors (all *p* < .001; see Supplementary Table S4 for means and SEs). The interaction of age group and channel for SO density (*F*(2,164.53) = 35.77, *p* <.001; Figure 2, A) showed higher density in infants than toddlers in frontal (*t*(221.58) = 7.82, *p* < .001) and central channels (*t*(221.58) = 3.50, *p* = .005), but not in parietal channels (*t*(221.58) = 0.14, *p* > .999). Infants’ SO density decreased from the frontal to the parietal channels (all *p* < .05). Contrarily, in toddlers, central and parietal channels showed greater SO density than frontal channels (all *p* < .002), with no difference between central and parietal channels (*p* > .999). The interaction between channel and age group for SO amplitude (*F*(2,163.59) = 9.89, *p* < .001; Figure 2, B), indicated higher amplitudes in toddlers than infants in all channels (all *p* < .007). Additionally, SO amplitude was smallest in parietal compared to frontal and central channels (all *p* < .001) in infants, with no difference between the frontal and central channels (*p* > .999). In toddlers, central channels showed higher amplitudes than frontal (*t*(170.83) = −3.14, *p* = .018) and parietal channels (*t*(170.83) = 5.89, *p* < .001). There was a trend for a higher amplitude in frontal compared to parietal channels (*t*(170.83) = 2.75, *p* = .059). The interaction of age group and channel for the SO slope (*F*(1,162.61) = 15.98, *p* < .001; Figure 2, C) demonstrated a steeper slope for each channel in toddlers than infants (all *p* < .001). Infants showed the smallest slope for parietal compared to frontal (*t*(170.74) = 4.53, *p < *.001) and central (*t*(170.74) = 5.86, *p* < .001) channels, with no difference between the frontal and central channels (*t*(170.74) = −1.33, *p* > .999). Toddlers had a steeper slope in parietal than frontal channels (*t*(170.74) = −3.22, *p* = .014), with both being flatter compared to central channels (all *p* < .005).

There was no interaction between age group and channel for SO duration (*F*(2,165.97) = 1.38, *p* = .253), but a main effect for each factor (Figure 2, D). SO duration was smallest at parietal channels compared to frontal (*t*(171) = 4.15, *p < *.001) and central channels (*t*(171) = 3.61, *p = *.001, main effect channel: *F*(2,165.97) = 10.47, *p* < .001), with no difference between the latter two (*t*(171) = 0.54, *p > *.999). Infants exhibited longer SOs than toddlers (*F*(1,233.8) = 146, *p* < .001).

Exploring whether the SO characteristics are due to the participants’ sleeping patterns, did not show a significant effect of the average number of naps per day on SO density (*p* = .683), amplitude (*p* = .575), slope (*p* = .780) or duration (*p* = .055). Although the covariate approached significance for sleep duration, the above-reported results stayed the same. Considering the homeostatic regulation of slow wave activity [37] and findings showing greater slow wave activity during evening than morning naps in 2-year-old children [38], we additionally explored whether the wake duration since the preceding nap and the timing of the experimental nap had an influence on the SO amplitude. Including these factors as a covariate left the described results unchanged and the covariates were not significant (all *p* > .560). Interestingly, when exploring potential differences between the first and last third of detected SOs, we found an interaction between the age group and third (*F*(1,400) = 5.71, *p* = .017) for the SO amplitude. While in infants the first and last third of detected SOs did not differ in their amplitude (*t*(410.3) = −0.29, *p > *.999), toddlers had higher amplitudes in the last third (*t*(410.3) = −3.96, *p < *.001). Again, the above-reported group × channel interaction effects remained unchanged. There were no effects on SO density and duration.

### Co-occurrence of SOs and spindles

**Figure 3.**
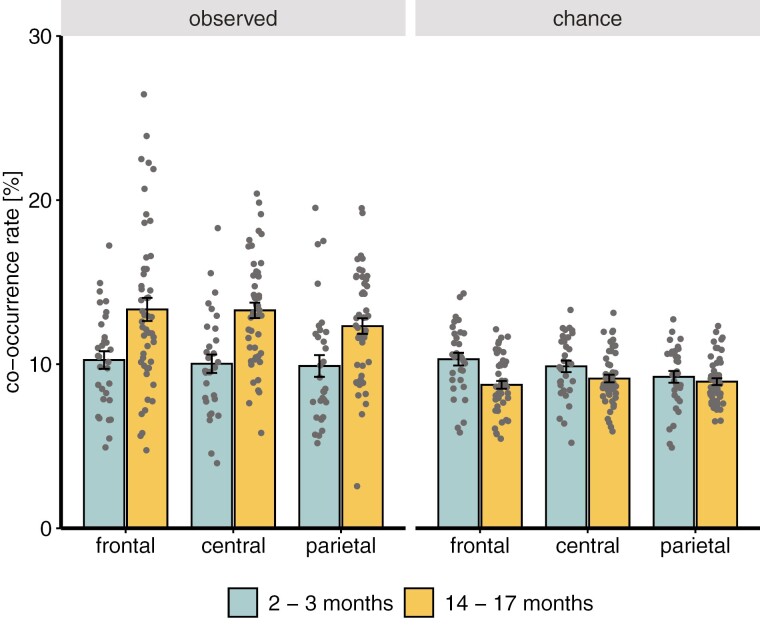
Slow oscillation–spindle co-occurrence dependent on age group and channel. Observed co-occurrence rates depict the percentage of spindles co-occurring with an SO between the two positive-to-negative zero crossings of an SO. Chance level represents the randomly expected proportion of spindles co-occurring with an SO, based on the proportion of non-REM sleep with SOs.

Analyses of co-occurrence rates revealed a significant main effect of age group, with age group being further involved in an interaction with co-occurrence type (*F*(1,406.72) = 108.78, *p* <.001). Bonferroni-corrected pairwise comparisons showed no difference between infants and toddlers regarding their co-occurrence rate expected by chance (*t*(345.78) = 2.00, *p* =.187). Furthermore, more spindles co-occurred with an SO in toddlers than infants (*t*(345.78) = −6.94, *p* <.001). Interestingly, while estimated co-occurrence rates exceeded chance level in toddlers (*t*(416.57) = 15.48, *p* <.001), this was not the case in infants (*t*(416.57) = 0.78, *p* >.999). There was no main effect of the channel (*F*(2,406.72) = 2.89, *p* =.057), or any other significant interactions (all *p* >.156; Figure 3; Supplementary Table S5). Furthermore, these results were not influenced by the participants’ average number of naps per day (*p* = .219).

### Peri-event time histograms

**Figure 4.**
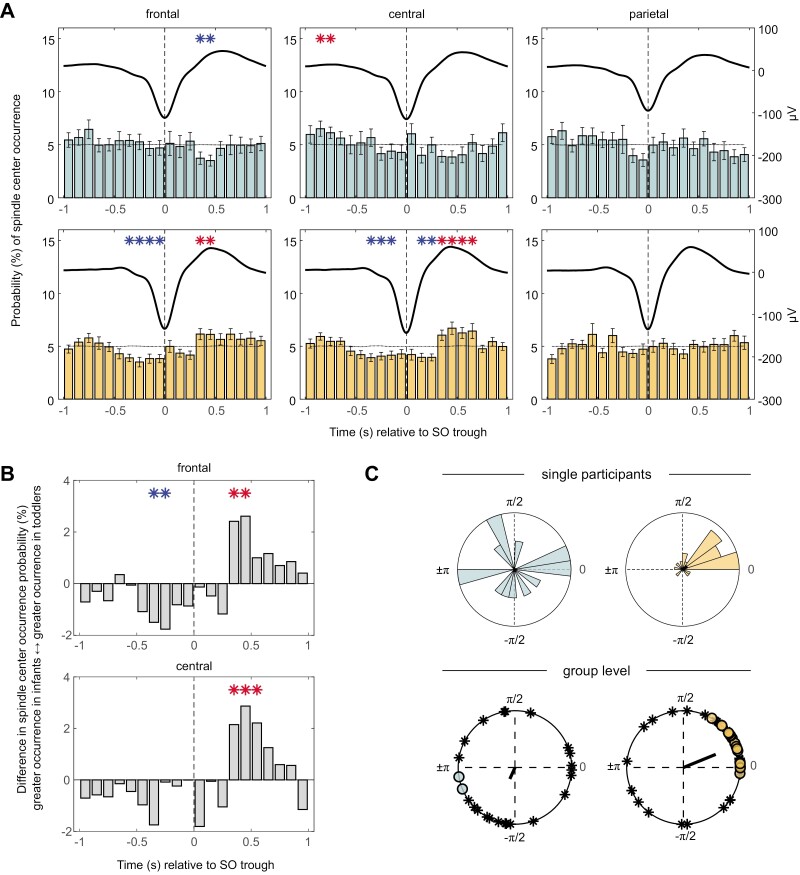
Slow oscillation–spindle coupling. (A) Peri-event time histograms (PETHs) of spindle center occurrence. Blue asterisks represent negative clusters and red asterisks positive clusters. Black lines depict the surrogate data plus the standard error. Average SOs are superimposed. (B) Difference in PETHs between toddlers and infants. Red asterisks indicate greater spindle center occurrence probabilities in toddlers than infants, while blue asterisks indicate lower probabilities in toddlers than infants. (C) Upper panel: polar histograms from 2 exemplary individuals in the age group 2–3 and 14–17 months, respectively. Lower panel: Mean preferred phases across participants. The direction of the vector depicts the circular group mean, with its length depicting the consistency of coupling within the age group. Filled circles represent participants with a non-uniform distribution of SO–spindle coupling while asterisks represent participants with uniform distribution.

While the analysis above already indicates that infants do not show above chance co-occurrence of spindles and SOs, the next set of analyses tries to answer the question, whether spindles tend to occur in a specific time window around the SO trough or whether they are spread evenly across the SO cycle for both age groups. Thus, as a second step, we analyzed the PETHs (Figure 4, A) and saw that while in infants, less spindles occurred frontally during the SO upstate (300–500 ms after the SO trough, cluster *p* = .002), more spindles occurred in that same time window in toddlers (cluster *p* = .013). Additionally, toddlers showed reduced spindle occurrence in the up-to-down state transition (−400 to 0 ms, *p* < .001) frontally. At central channels, infants showed increased spindle occurrence between −900 and −700 ms prior to the SO downstate (cluster *p* = .010). Interestingly, toddlers again showed increased spindle occurrence during the SO upstate (300–700 ms, cluster *p* < .001) but not during the SO trough. The SO transitions were marked by a decreased occurrence in both, the up-to-down state (cluster *p* < .001) and the down-to-up state (cluster *p* = .001). In both infants and toddlers, spindles were distributed equally in the ±1000 ms around the SO trough at parietal channels. Directly comparing PETHs of toddlers and infants showed that toddlers had a reduced spindle center occurrence probability during the up-to-down state transition compared to infants for frontal channels (−400 to −200 ms, cluster *p* = .008), but increased occurrence probability during the SO upstate (300–500 ms after the SO trough, cluster *p* < .001). Similarly, the difference between toddlers and infants in spindle center occurrence during the SO upstate in the central channels was significant (300–700 ms, cluster *p* < .001; Figure 4, B). There was no difference between the two age groups at parietal channels.

### Time-frequency representations

**Figure 5.**
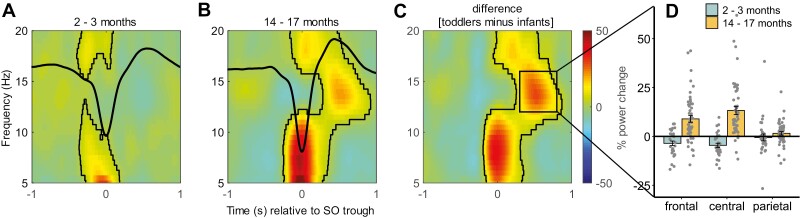
Time-frequency representations (TFRs) of SO events in infants and toddlers in the central channel. TFRs of the central channel are depicted for infants (A) and toddlers (B). Plots depict the change from baseline (in %). Black lines indicate the outline of significant positive clusters. Warm colors indicate greater power compared to baseline. (C) Difference between toddlers and infants in the change from baseline, with warmer colors indicating greater changes in toddlers than infants. (D) Change from baseline averaged across the spindle frequency range (12–16 Hz) during the SO upstate (300–800 ms), separately for infants and toddlers and frontal, central, and parietal channels.

A similar picture emerged, testing baseline-normalized SO-locked TFRs against zero (see Methods for details). Both age groups showed an increase in power at the SO trough in lower frequencies around 5–12 Hz in all channels (all clusters *p* < .001; Figure 5, A and B). Only in toddlers, these positive clusters extended to further frequencies and time windows. Specifically, toddlers showed an increase in power in the spindle frequency range (11–16 Hz) during the SO upstate (~250–1000 ms) in frontal and central channels and again during the SO trough an increase in higher frequencies from 16 to 20 Hz. Additionally, infants showed reduced activity between 10 and 18 Hz around the SO upstate (~100–600 ms) in frontal (cluster *p* = .006) and central channels (cluster *p* < .001). When directly comparing baseline-normalized TFRs of infants and toddlers (Figure 5, C), we saw that these changes from baseline were bigger in toddlers than infants. Firstly, the increase in 5–12 Hz power during the SO trough, which all participants showed, was greater in toddlers than infants in all channels (frontal and central cluster *p* < .001, parietal cluster *p* = .017). Again, in frontal and central channels this cluster spread to further frequency and time ranges. The increase in spindle power during the SO upstate was indeed greater in toddlers than infants. Similarly, the increase in the higher frequencies (>15 Hz) during the SO downstate was greater in toddlers than infants. To explicitly test topographical differences regarding spindle activity during the SO upstate, spindle power (12–16 Hz) during the SO upstate (300–800 ms) was averaged for each individual in each channel and then subjected to a linear mixed-effects model. A significant age group × channel interaction (*F*(2,164.75) = 14.22, *p* < .001; Figure 5, D) indicated that toddlers show greater spindle activity during the SO upstate than infants in all (all *p* < .001) but the parietal channel (*p* > .999). Infants did not exhibit any channel differences regarding the changes in power from baseline (all *p* > .827). On the other hand, toddlers showed smaller changes from baseline in spindle activity during the SO upstate in the parietal channel compared to the frontal (*t*(171.27) = 3.92, *p* = .001) and central channels (*t*(171.27) = 6.18, *p* < .001), with no difference between the latter two (*t*(171.27) = −2.26, *p* = .226).

### Phase-amplitude coupling

The picture that SOs and spindles are not yet coupled in infants and only start to coincide in specific regions in toddlers similarly emerges regarding the uniformity in the coupling of spindle amplitude maxima across the SO cycle (Rayleigh Test). For the following analyses, only the channels of an individual with at least five co-occurring events were included, leading to reduced sample sizes, especially in infants (infants: *n* = 22–27, toddlers: *n* = 42–47). None of the infants showed a nonuniform distribution of SO–spindle coupling at frontal channels. At central and parietal channels, two and three infants showed a nonuniform distribution respectively. In toddlers, SO–spindle coupling in parietal channels was mostly uniformly distributed (one and three children with a nonuniform distribution). At frontal channels, up to 16 toddlers and at central channels, up to 18 toddlers had a nonuniform distribution. Further analyses were restricted to channel Cz due to being the channel with the most available datapoints (i.e. most participants ≥ 5 co-occurring events) that were nonuniformly distributed. The two age groups did not differ significantly in how consistently spindles are clustered toward the same SO phase (*F*(1,56.63) = 2.45, *p* = .123, infants: M = .29, SEM = .03; toddlers: M = .36, SEM = .03). However, the two groups differed in their mean preferred phase of coupling (Whatson-Wheeler test, *W*(2) = 16.56, *p* < .001, infants: $\bar{\theta }$ = −114°, toddlers: $\bar{\theta }$ = 22°). Results regarding the mean preferred phase of coupling should be interpreted with caution, since most participants exhibited uniformly distributed phase-amplitude coupling, limiting the interpretability of the calculated circular mean. Computing a Rayleigh test across the mean preferred phases within an age group, showed a uniform distribution of mean preferred phases in infants (Rayleigh-Z = 1.17, *p* = .313) and a non-uniform distribution in toddlers (Rayleigh-Z = 17.64, *p* < .001; Figure 4, C).

### Spindle and SO characteristics of (non) co-occurring events

In the last step, we exploratively analyzed whether co-occurring and non-co-occurring spindles and SOs differ in their characteristics. Spindle amplitude and frequency did not differ between those spindles that co-occur with an SO and those that did not co-occur with an SO (all *p* > .37, see also Supplementary Figure S1). Interestingly, the factor type (co-occurring vs. non-co-occurring) was involved in an interaction with the age group for all SO characteristics (amplitude, slope, and duration). Including this factor did not change the results of age group and channel effects reported above and is, therefore, not reported again. Furthermore, none of the models showed a significant three-way interaction between age group × channel × type (all *p* > 145). The interaction of age group × type (*F*(1,404.19) = 12.52, *p* < .001) revealed in infants no difference in amplitude of co-occurring and non-co-occurring SOs (*t*(417) = 0.095, *p* > .999). In toddlers, amplitudes of co-occurring SOs (M = 259 µV, SEM = 4.30) were higher than those of non-co-occurring SOs (M = 247 µV, SEM = 3.91, *t*(417) = 5.73, *p* < .001). For both, co-occurring and non-co-occurring SOs, toddlers showed greater amplitudes than infants (all *p* < .001). The same pattern of results emerged for the SO slope (age group × type interaction: *F*(1,402.40) = 17.15, *p* < .001). Interestingly, for SO duration the interaction between age group and type (*F*(1,405.84) = 4.92, *p* = .027), indicated that while for both age groups, the co-occurring SOs were longer than the non-co-occurring SOs, the duration difference between co-occurring and non-co-occurring events was greater in infants (M_diff_ = 0.07, SEM = 0.01) than toddlers (M_diff_ = 0.04, SEM = 0.01; Supplementary Figure S2).

## Discussion

Comparing daytime nap recordings between a group of infants (2–3 months) and toddlers (14–17 months), we observed a clear pattern of differences in SOs, spindles, and their temporal coupling between the groups, indicative of early brain maturation. While spindles and SOs each displayed their distinct maturational changes, an adult-like coupling of spindles to the upstate of SOs was only observed in toddlerhood, whereas such coupling was absent in the infants. Indeed, in the infants, the occurrence of spindles over the frontal cortex appeared to be even decreased during the SO upstate. The findings suggest an immature, and possibly less effective, processing of memory information in corticothalamic networks during sleep in early infancy.

Spindles, among the oscillatory markers of sleep, have been found to exhibit the most profound changes within the first year of life [9]. While this study by Kwon et al. [9] did not reveal a difference in spindle density between 1- and 2-year-olds, in the present study spindle density in toddlers was lower than in infants, possibly reflecting an ongoing decrease in spindle rate from 4 months onwards [9, 39]. Spindle density did not differ between recording sites in our infants. Toddlers, however, opposite to the adult-like pattern, exhibited lower density in the parietal than frontal or central areas. This topographical pattern of maximum spindle density is in line with a recent study in 6-month-old infants using high-density EEG [40]. A comparison of 12–19 with 22- to 30-month-old participants suggests that spindle density at parietal areas continues to decrease [41]. Similarly, spindle amplitude was lower at parietal than frontal or central areas in both age groups in our study. Previous findings on sigma power revealed no age-dependent differences along the anteroposterior axis in a sample of children aged between 0 and 48 months [42]. However, several of our analyzed EEG oscillatory parameters (e.g. spindle amplitude, SO density, as well as spindle power during the SO upstate) indicated the lowest activity in parietal sites in toddlers. Moreover, while the age groups clearly differed in activity recorded from frontal and central channels in these measures, this was not the case for parietal recordings. Overall, the predominance of the changes over anterior regions may reflect more dynamic maturational changes, related, e.g., to myelinization and synaptogenesis, in frontal cortical networks than in the posterior cortex in this age range [43].

Similar to Fattinger et al. [12] reporting an increase in the SO slope within the first 9 months of life, we found a steeper slope in toddlers than infants in all channels. Contrary to the present findings, in that study, the slope did not differ between frontal, central, and parietal sites, but was steepest at occipital sites. In the present study, the topography for the maximum SO slope depended on the age group: infants displayed the steepest slope fronto-centrally, which concurs with findings in 6-month-olds displaying the steepest slope at frontal sites, but also at occipital sites [18]. In contrast, our toddlers, although showing generally steeper slopes than the infants, displayed relatively smaller slope values for the frontal than central sites, which fits with previous research in somewhat older children, suggesting that slow oscillatory activity matures along the posteroanterior axis from the age of 2 years onwards [8]. In combination with the present findings, these studies point to a u-shaped developmental trajectory for the slope of frontal cortical SOs, such that relative maximum slopes in this region are reached during early infancy as well as early childhood whereas toddlerhood is characterized by a minimum in the frontal SO slope relative to more posterior regions.

The slope of the SO primarily reflects the extent of synchronization in the underlying neuronal membrane potentials and has been proposed as an indicator of synaptic strength [44, 45]. During development, the extent of synchronization may also originate from changes in synaptogenesis. Accordingly, the general increase in SO slope across our two age groups, in combination with the relative minimum of slope values seen in the toddlers’ frontocortical recordings, may reflect an enhanced synaptogenesis in this age range which is somewhat more pronounced over posterior than frontal cortical areas. This interpretation, however, remains tentative, also in light of the fact that it is currently unclear to what extent the slow oscillatory slope is similarly tightly linked to processes of synaptic pruning that dominate later childhood and adolescence [46–49].

The main target of our investigation was the co-occurrence of SOs and spindles. We show, for the first time, that co-occurrence rates do not exceed above what is expected by chance in infants, but are significantly increased at frontocentral recording sites only in toddlerhood. This finding is even more interesting against the backdrop that our infants had longer SOs than toddlers, and additionally a higher density of SOs and spindles. Thus, although in infants the chance that a spindle coincides with a SO was descriptively a priori higher than in toddlers (difference not significant), the actual co-occurrence rate observed in the infants was significantly smaller than in toddlers. In light of findings by Jaramillo et al. [18] indicating a temporal coupling of slow waves and spindles in 6-month-old infants, with maximal coupling strength over the occipital cortex, we cannot exclude that a significant SO–spindle co-occurrence at these most posterior cortical sites was also present in our 3-month-old infants, as our electrode arrangement did not cover the occipital cortex. However, this possibility seems unlikely, given that there was not even a frontal-to-parietal topographical trend toward an enhanced SO–spindle co-occurrence at posterior cortical sites at a descriptive level (Figure 3). Beyond this, the direct comparison with the findings by Jaramillo et al. [18] is hampered by methodological differences between the present and the foregoing study. Importantly, Jaramillo et al. [18] did not assess the chance level of SO–spindle co-occurrence at the occipital electrode sites and used a wider ±2.5-second (rather than ±1.0 second) window around the SO trough to determine the co-occurrence of a spindle event. This resulted in a co-occurrence rate of almost 63% at occipital sites, which strikingly differs from the average co-occurrence rate of 10% at frontal, central, and parietal sites in the younger infants of the present study (for these more anterior sites, co-occurrence rates are not reported in Jaramillo et al. [18]).

Strikingly, examining PETHs we observed significantly reduced spindle occurrence during the SO upstate at frontal sites in our infant group (compared to averaged bin-shuffled surrogate data, Figure 4). Contrarily, our toddlers showed greater spindle center occurrence during the SO upstate at the frontal as well as central recording site. This pattern was similarly seen in TFRs of SO events, where again, infants presented reduced spindle power around the SO upstate, while toddlers showed increased spindle power during the upstate in frontal and central channels (Figure 5). A related difference between the age groups was revealed by exploratory analyses of phase-amplitude coupling between SOs and spindles (which, due to the low co-occurrence rates particularly in the infants, were only performed for the central channel). In toddlers, like in adults, spindles coupled (with the “mean preferred phase”) to the SO upstate, whereas in infants the preferred phase of coupling of spindles seemed to be toward the SO downstate. Overall, these differences focusing on frontal-central recording sites, suggest particularly strong maturational dynamics in thalamo-frontocortical networks during this age interval, with the adult-like pattern of more posterior spindles nesting into the upstate of co-occurring SOs, beginning to emerge only at toddlerhood. In the 2- to 3-month-old infants, on the other hand, a precise nesting of spindles into the SO upstate is lacking and, if at all, a seemingly immature form of the interplay between these oscillatory events is established in anterior thalamocortical networks, with potentially reduced effectiveness in processing memory.

Whether the lack of temporal coordination between SOs and spindles in the 2- to 3-month-old infants is of primary cortical or thalamic origin, cannot be answered. Noteworthy in this context is a study in young school kids (5–11 years) who showed the classical pattern of an increased spindle co-occurrence during the SO upstate only for spindles detected in the “adult-like” 12.5–16 Hz frequency range, but no co-occurrence when spindles, as in the present study, were identified around the individually determined frequency peak in the power spectrum which tended to be at somewhat slower frequencies in the young school children [10]. Indeed, identifying spindles with reference to individual frequency peaks in the EEG power spectrum reveals a rather complex developmental pattern. Simply put, initially during infancy, spindles are clustered around one spectral peak at a rather fast frequency [9]. During toddlerhood, the single spectral spindle peak often becomes replaced by two separate spectral peaks, representing slow vs. fast spindles with the frequency of the fast spindle peak being still slightly slower than that of the spindle peak during infancy. Across childhood and adolescence, fast spindles again increase in frequency and develop a more distinct centro-parietal topography, whereas the slow spindles become more restricted to frontal recording sites [9]. According to this developmental course, the toddlers (14–17 months) and infants of our study are at an age for which a clear separation into slow frontal and fast central spindles only starts to emerge. Indeed, only the minority (~9% of the infants and ~13% of the toddlers) of participants exhibited two clearly discernible frequency peaks in the spindle range in the power spectrum. It is conceivable, however, that the single spectral peak we used for spindle detection in our groups, represents developmental precursor spindles of mixed quality, i.e. those developing into classical fast spindles and nest into the SO upstate, and those developing into slow spindles and at adulthood, typically occur at the transition into the SO downstate [26, 50]. Ultimately, the view of immature spindle generation as a primary factor underlying the lack of SO–spindle synchronization during infancy is also supported by experiments in rat pups, indicating that the phase-coupling of hippocampal ripples to spindles is likewise impaired during early development [51].

A limitation of our study is the cross-sectional design, particularly in light of the rather complex developmental course of spindles in this age range. Longitudinal assessments should allow for individually following the putative branching of precursor spindles as present in infancy into fast and slow types of spindles during early childhood. Additionally, we report findings from daytime naps only. It has previously been shown that spindle characteristics differ between daytime nap and nighttime sleep recordings [52, 53]. However, the two age groups we examined differ in their sleep-wake rhythm. While most of the toddlers in our study showed consolidated periods of wakefulness and sleep with an average of ~ one nap per day, daytime wakefulness was still quite fragmented in the infants and the circadian system still continues to mature [54]. To explore whether our findings might be influenced by the sleep-wake patterns, we included the average number of naps during the day as a covariate. This left our results basically unchanged. Nonetheless, it might be possible that circadian effects expressed by different measures might lead to different results. Since the infant group is still developing their sleep-wake patterns, it might also be the case that only the toddlers in our study are affected by circadian rhythmicity. This is also reflected by our explorative analysis showing only in toddlers a decline in SO amplitudes from the first to the last third of detected events (cf. [55]).

Another limitation of our study is that we did not relate our sleep EEG findings to any performance measure of memory. In the next step, such analyses will reveal whether the emergence of a coordinate coalescence of spindles into SO upstates in toddlers is associated with an enhanced memory consolidation. However, it is also important to note here that there is some evidence suggesting that sleep supports memory formation already at an age before toddlerhood [19, 56–58]. At this early age, spindles alone may suffice to produce such memory benefits. In sum, our study is the first to show that a nesting of spindles into SO upstates is absent in early infancy. Future studies are warranted to examine how this immaturity relates to the infant’s capabilities to use sleep for memory formation.

## Supplementary Material

zpae084_suppl_Supplementary_Material

## Data Availability

The data underlying this article will be shared on request to the corresponding author.

## References

[1] Nir Y , Staba Richard J, Andrillon T, et al Regional slow waves and spindles in human sleep. Neuron. 2011;70(1):153–169. doi: https://doi.org/10.1016/j.neuron.2011.02.043 21482364 PMC3108825

[2] Steriade M , Timofeev I, Grenier F. Natural waking and sleep states: a view from inside neocortical neurons. J Neurophysiol. 2001;85(5):1969–1985. doi: https://doi.org/10.1152/jn.2001.85.5.1969 11353014

[3] Steriade M. Coherent oscillations and short-term plasticity in corticothalamic networks. Trends Neurosci. 1999;22(8):337–345. doi: https://doi.org/10.1016/s0166-2236(99)01407-1 10407416

[4] Diekelmann S , Born J. The memory function of sleep. Nat Rev Neurosci. 2010;11(2):114–126. doi: https://doi.org/10.1038/nrn2762 20046194

[5] Niethard N , Ngo H-VV, Ehrlich I, Born J. Cortical circuit activity underlying sleep slow oscillations and spindles. Proc Natl Acad Sci USA. 2018;115(39):E9220–E9229. doi: https://doi.org/10.1073/pnas.1805517115 30209214 PMC6166829

[6] Brodt S , Inostroza M, Niethard N, Born J. Sleep-A brain-state serving systems memory consolidation. Neuron. 2023;111(7):1050–1075. doi: https://doi.org/10.1016/j.neuron.2023.03.005 37023710

[7] Peyrache A , Seibt J. A mechanism for learning with sleep spindles. Philos Trans R Soc Lond B Biol Sci. 2020;375(1799):20190230. doi: https://doi.org/10.1098/rstb.2019.0230 32248788 PMC7209910

[8] Kurth S , Ringli M, Geiger A, LeBourgeois M, Jenni OG, Huber R. Mapping of cortical activity in the first two decades of life: a high-density sleep electroencephalogram study. J Neurosci. 2010;30(40):13211–13219. doi: https://doi.org/10.1523/JNEUROSCI.2532-10.2010 20926647 PMC3010358

[9] Kwon H , Walsh KG, Berja ED, et al Sleep spindles in the healthy brain from birth through 18 years. Sleep. 2023;46(4). doi: https://doi.org/10.1093/sleep/zsad017 PMC10091086 36719044

[10] Joechner A-K , Hahn MA, Gruber G, Hoedlmoser K, Werkle-Bergner M. Sleep spindle maturity promotes slow oscillation-spindle coupling across child and adolescent development. eLife. 2023;12:e83565. doi: https://doi.org/10.7554/eLife.83565 37999945 PMC10672804

[11] Kurz E-M , Zinke K, Born J. Sleep electroencephalogram (EEG) oscillations and associated memory processing during childhood and early adolescence. Dev Psychol. 2023;59(2):297–311. doi: https://doi.org/10.1037/dev0001487 36395048

[12] Fattinger S , Jenni OG, Schmitt B, Achermann P, Huber R. Overnight changes in the slope of sleep slow waves during infancy. Sleep. 2014;37(2):245–253. doi: https://doi.org/10.5665/sleep.3390 24497653 PMC3900623

[13] Bastian L , Samanta A, Ribeiro de Paula D, et al Spindle–slow oscillation coupling correlates with memory performance and connectivity changes in a hippocampal network after sleep. Hum Brain Mapp. 2022;43(13):3923–3943. doi: https://doi.org/10.1002/hbm.25893 35488512 PMC9374888

[14] Contreras MP , Fechner J, Born J, Inostroza M. Accelerating maturation of spatial memory systems by experience: evidence from sleep oscillation signatures of memory processing. J Neurosci. 2023;43(19):3509–3519. doi: https://doi.org/10.1523/JNEUROSCI.1967-22.2023 36931711 PMC10184732

[15] Hahn M , Heib D, Schabus M, Hoedlmoser K, Helfrich RF. Slow oscillation-spindle coupling predicts enhanced memory formation from childhood to adolescence. eLife. 2020;9:e53730. doi: https://doi.org/10.7554/eLife.53730 32579108 PMC7314542

[16] Helfrich RF , Mander BA, Jagust WJ, Knight RT, Walker MP. Old brains come uncoupled in sleep: slow wave-spindle synchrony, brain atrophy, and forgetting. Neuron. 2018;97(1):221–230.e4. doi: https://doi.org/10.1016/j.neuron.2017.11.020 29249289 PMC5754239

[17] Klinzing JG , Niethard N, Born J. Mechanisms of systems memory consolidation during sleep. Nat Neurosci. 2019;22(10):1598–1610. doi: https://doi.org/10.1038/s41593-019-0467-3 31451802

[18] Jaramillo V , Schoch SF, Markovic A, et al An infant sleep electroencephalographic marker of thalamocortical connectivity predicts behavioral outcome in late infancy. Neuroimage. 2023;269:119924. doi: https://doi.org/10.1016/j.neuroimage.2023.119924 36739104

[19] Friedrich M , Mölle M, Born J, Friederici AD. Memory for nonadjacent dependencies in the first year of life and its relation to sleep. Nat Commun. 2022;13(1):7896. doi: https://doi.org/10.1038/s41467-022-35558-x 36550131 PMC9780241

[20] Grigg-Damberger M , Gozal D, Marcus CL, et al The visual scoring of sleep and arousal in infants and children. J Clin Sleep Med. 2007;03(02):201–240. doi: https://doi.org/10.5664/jcsm.26819 17557427

[21] Grigg-Damberger MM. The visual scoring of sleep in infants 0 to 2 months of age. J Clin Sleep Med. 2016;12(3):429–445. doi: https://doi.org/10.5664/jcsm.5600 26951412 PMC4773630

[22] Donoghue T , Haller M, Peterson EJ, et al Parameterizing neural power spectra into periodic and aperiodic components. Nat Neurosci. 2020;23(12):1655–1665. doi: https://doi.org/10.1038/s41593-020-00744-x 33230329 PMC8106550

[23] Ostlund B , Donoghue T, Anaya B, et al Spectral parameterization for studying neurodevelopment: how and why. Dev Cogn Neurosci. 2022;54:101073. doi: https://doi.org/10.1016/j.dcn.2022.101073 35074579 PMC8792072

[24] Mölle M , Marshall L, Gais S, Born J. Grouping of spindle activity during slow oscillations in human non-rapid eye movement sleep. J Neurosci. 2002;22(24):10941–10947. doi: https://doi.org/10.1523/JNEUROSCI.22-24-10941.2002 12486189 PMC6758415

[25] Kurth S , Jenni OG, Riedner BA, Tononi G, Carskadon MA, Huber R. Characteristics of sleep slow waves in children and adolescents. Sleep. 2010;33(4):475–480. doi: https://doi.org/10.1093/sleep/33.4.475 20394316 PMC2849786

[26] Mölle M , Bergmann TO, Marshall L, Born J. Fast and slow spindles during the sleep slow oscillation: disparate coalescence and engagement in memory processing. Sleep. 2011;34(10):1411–1421. doi: https://doi.org/10.5665/SLEEP.1290 21966073 PMC3174843

[27] Muehlroth BE , Sander MC, Fandakova Y, et al Precise slow oscillation-spindle coupling promotes memory consolidation in younger and older adults. Sci Rep. 2019;9(1):1940. doi: https://doi.org/10.1038/s41598-018-36557-z 30760741 PMC6374430

[28] Oostenveld R , Fries P, Maris E, Schoffelen JM. FieldTrip: open source software for advanced analysis of MEG, EEG, and invasive electrophysiological data. Comput Intell Neurosci. 2011;2011:156869. doi: https://doi.org/10.1155/2011/156869 21253357 PMC3021840

[29] Denis D , Mylonas D, Poskanzer C, Bursal V, Payne JD, Stickgold R. Sleep spindles preferentially consolidate weakly encoded memories. J Neurosci. 2021;41(18):4088–4099. doi: https://doi.org/10.1523/JNEUROSCI.0818-20.2021 33741722 PMC8176750

[30] Berens PC. A MATLAB toolbox for circular statistics. J Stat Softw. 2009;31(10):21. doi: https://doi.org/10.18637/jss.v031.i10

[31] R Core Team. R: a language and environment for statistical computing. Vienna, Austria: Foundation for Statistical Computing; 2023.

[32] Bates D , Mächler M, Bolker B, Walker S. Fitting linear mixed-effects models using lme4. J Stat Softw. 2015;67:1–48. doi: https://doi.org/10.18637/jss.v067.i01

[33] Kuznetsova A , Brockhoff PB, Christensen RH. lmerTest package: tests in linear mixed effects models. J Stat Softw. 2017;82(13):1–26.

[34] Lenth RV. emmeans: estimated marginal means, aka least-squares means. R package version 1.9.0. 2023. https://CRAN.R-project.org/package=emmeans

[35] Maris E , Oostenveld R. Nonparametric statistical testing of EEG- and MEG-data. J Neurosci Methods. 2007;164(1):177–190. doi: https://doi.org/10.1016/j.jneumeth.2007.03.024 17517438

[36] Agostinelli C , Lund U. R package “circular”: circular statistics. version 0.5-0. 2023. https://CRAN.R-project.org/package=circular

[37] Borbély AA , Baumann F, Brandeis D, Strauch I, Lehmann D. Sleep deprivation: effect on sleep stages and EEG power density in man. Electroencephalogr Clin Neurophysiol. 1981;51(5):483–495. doi: https://doi.org/10.1016/0013-4694(81)90225-x 6165548

[38] Kurth S , Lassonde JM, Pierpoint LA, et al Development of nap neurophysiology: preliminary insights into sleep regulation in early childhood. J Sleep Res. 2016;25(6):646–654. doi: https://doi.org/10.1111/jsr.12427 27252144 PMC5135687

[39] D’Atri A , Novelli L, Ferrara M, Bruni O, De Gennaro L. Different maturational changes of fast and slow sleep spindles in the first four years of life. Sleep Med. 2018;42:73–82. doi: https://doi.org/10.1016/j.sleep.2017.11.1138 29458750

[40] Schoch SF , Jaramillo V, Markovic A, et al Bedtime to the brain: how infants’ sleep behaviours intertwine with non-rapid eye movement sleep electroencephalography features. J Sleep Res. 2024;33(2):e13936. doi: https://doi.org/10.1111/jsr.13936 37217191

[41] Page J , Lustenberger C, Frӧhlich F. Social, motor, and cognitive development through the lens of sleep network dynamics in infants and toddlers between 12 and 30 months of age. Sleep. 2018;41(4). doi: https://doi.org/10.1093/sleep/zsy024 PMC6018907 29506060

[42] Novelli L , D’atri A, Marzano C, et al Mapping changes in cortical activity during sleep in the first 4 years of life. J Sleep Res. 2016;25(4):381–389. doi: https://doi.org/10.1111/jsr.12390 26854271

[43] Hodel AS. Rapid infant prefrontal cortex development and sensitivity to early environmental experience. Dev Rev. 2018;48:113–144. doi: https://doi.org/10.1016/j.dr.2018.02.003 30270962 PMC6157748

[44] Riedner BA , Vyazovskiy VV, Huber R, et al Sleep homeostasis and cortical synchronization: III. A high-density EEG study of sleep slow waves in humans. Sleep. 2007;30(12):1643–1657. doi: https://doi.org/10.1093/sleep/30.12.1643 18246974 PMC2276133

[45] Vyazovskiy VV , Olcese U, Lazimy YM, et al Cortical firing and sleep homeostasis. Neuron. 2009;63(6):865–878. doi: https://doi.org/10.1016/j.neuron.2009.08.024 19778514 PMC2819325

[46] Buchmann A , Ringli M, Kurth S, et al EEG sleep slow-wave activity as a mirror of cortical maturation. Cerebral cortex 2011;21(3):607–615. doi: https://doi.org/10.1093/cercor/bhq129 20624840

[47] Feinberg I , Campbell IG. Sleep EEG changes during adolescence: an index of a fundamental brain reorganization. Brain Cogn. 2010;72(1):56–65. doi: https://doi.org/10.1016/j.bandc.2009.09.008 19883968

[48] Huttenlocher PR. Synaptic density in human frontal cortex - developmental changes and effects of aging. Brain Res. 1979;163(2):195–205. doi: https://doi.org/10.1016/0006-8993(79)90349-4 427544

[49] Huttenlocher PR , Dabholkar AS. Regional differences in synaptogenesis in human cerebral cortex. J Comp Neurol. 1997;387(2):167–178. doi: https://doi.org/10.1002/(sici)1096-9861(19971020)387:2<167::aid-cne1>3.0.co;2-z 9336221

[50] Staresina BP , Bergmann TO, Bonnefond M, et al Hierarchical nesting of slow oscillations, spindles and ripples in the human hippocampus during sleep. Nat Neurosci. 2015;18(11):1679–1686. doi: https://doi.org/10.1038/nn.4119 26389842 PMC4625581

[51] Fechner J , Contreras MP, Zorzo C, Shan X, Born J, Inostroza M. Sleep slow oscillation-spindle coupling precedes spindle-ripple coupling during development. Sleep. 2024;47. doi: https://doi.org/10.1093/sleep/zsae061 38452190

[52] Bódizs R , Horváth CG, Szalárdy O, et al Sleep-spindle frequency: overnight dynamics, afternoon nap effects, and possible circadian modulation. J Sleep Res. 2022;31(3):e13514. doi: https://doi.org/10.1111/jsr.13514 34761463

[53] Knoblauch V , Münch M, Blatter K, et al Age-related changes in the circadian modulation of sleep-spindle frequency during nap sleep. Sleep. 2005;28(9):1093–1101. doi: https://doi.org/10.1093/sleep/28.9.1093 16268378

[54] Rivkees SA. Developing circadian rhythmicity in infants. Pediatrics. 2003;112(2):373–381. doi: https://doi.org/10.1542/peds.112.2.373 12897290

[55] Jenni OG , Borbely AA, Achermann P. Development of the nocturnal sleep electroencephalogram in human infants. Am J Physiol Regul Integr Comp Physiol. 2004;286(3):R528–R538. doi: https://doi.org/10.1152/ajpregu.00503.2003 14630625

[56] Friedrich M , Wilhelm I, Molle M, Born J, Friederici AD. The sleeping infant brain anticipates development. Curr Biol. 2017;27(15):2374–2380.e3. doi: https://doi.org/10.1016/j.cub.2017.06.070 28756948

[57] Konrad C , Herbert JS, Schneider S, Seehagen S. Gist extraction and sleep in 12-month-old infants. Neurobiol Learn Mem. 2016;134 Pt B(Pt B):216–220. doi: https://doi.org/10.1016/j.nlm.2016.08.021 27587286

[58] Horváth K , Hannon B, Ujma PP, Gombos F, Plunkett K. Memory in 3-month-old infants benefits from a short nap. Dev Sci. 2018;21(3):e12587. doi: https://doi.org/10.1111/desc.12587 28722249

