# Supplementary material for: Development of slow oscillation–spindle coupling from infancy to toddlerhood
Source: Sleep Adv. 2024 Nov 16;5(1):zpae084. doi: 10.1093/sleepadvances/zpae084 (PMC11630081; doi:10.1093/sleepadvances/zpae084)
Supplement: zpae084_suppl_Supplementary_Material [file zpae084_suppl_supplementary_material.docx]

**Supporting Information**

**Development of Slow Oscillation-Spindle Coupling from Infancy to Toddlerhood**

Eva-Maria Kurz^1,2$^, Lisa Bastian^1,3$^, Matthias Mölle^4^, Jan Born^1,5,6^, Manuela Friedrich^7,8^

^1^Institute of Medical Psychology and Behavioral Neurobiology, University of Tübingen, 72076

Tübingen, Germany, DE

^2^Department of Child and Adolescent Psychiatry, Psychosomatics and Psychotherapy, University

Hospital of Psychiatry and Psychotherapy, University of Tübingen, 72076 Tübingen, Germany,

DE

^3^Max Planck School of Cognition, Max Planck Institute for Human Cognitive and Brain Sciences,

04103 Leipzig, Germany, DE

^4^Center of Brain, Behavior and Metabolism (CBBM), University of Lübeck, 23562 Lübeck, Germany, DE

^5^Center for Integrative Neuroscience, University of Tübingen, 72076 Tübingen, Germany

^6^German Center for Mental Health (DZPG), site Tübingen, Germany

^7^Department of Psychology, Humboldt-University, 12489 Berlin, Germany, DE

^8^Department of Neuropsychology, Max Planck Institute for Human Cognitive and Brain Sciences,

04103 Leipzig, Germany, DE

$ shared first authorship

**Table S1**

Sleep Diary

|  | **2-3 months** (*N* = 28) | |  | **14-17 months**  (*N* = 47) | |  | **Group comparison** |
| --- | --- | --- | --- | --- | --- | --- | --- |
|  | ***Mean (SEM)*** | **range** |  | ***Mean (SEM)*** | **range** |  | ***p*** |
| **TST day**  **(hh:mm)** | **04:25 (00:22)** | **02:05-13:45** |  | **01:50 (00:03)** | **00:33-02:40** |  | **< .001** |
| TST night  (hh:mm) | 10:16 (09:18) | 08:24-11:27 |  | 10:32 (00:06) | 09:02-11:47 |  | .159 |
| **# naps day** | **3.83 (0.90)** | **1.80-5.14** |  | **1.32 (0.32)** | **0.86-2.14** |  | **< .001** |
| **# awakening night** | **2.54 (1.26)** | **0-5.29** |  | **1.98 (1.56)** | **0-5.86** |  | **.040** |

**Table S2**

Non-REM sleep characteristics

|  | **2-3 months** (*N* = 31) | |  | **14-17 months**  (*N* = 49) | |  | **Group comparison** |
| --- | --- | --- | --- | --- | --- | --- | --- |
|  | ***Mean (SEM)*** | **range** |  | ***Mean (SEM)*** | **range** |  | ***p*** |
| Sleep Onset (hh:mm) | 13:18 (00:17) | 10:34-17:21 |  | 13:29 (00:13) | 09:51-18:25 |  | .502 |
| **TST** | **50.31 (3.78)** | **22.00-107.00** |  | **62.16 (3.25)** | **23.50-127.50** |  | **.009** |
| N2 (min) | 22.50 (2.02) | 6.50-49.50 |  | 26.73 (1.65) | 9.50-54.50 |  | .089 |
| **N3 (min)** | **9.69 (1.57)** | **0.00-32.50** |  | **19.92 (1.58)** | **0.00-46.50** |  | **< .001** |
| N2 (%) | 44.89 (3.21) | 16.05-88.89 |  | 43.47 (2.08) | 16.08-75.20 |  | .694 |
| **N3 (%)** | **20.18 (3.26)** | **0.00-76.54** |  | **32.05 (2.19)** | **0.00-59.30** |  | **.003** |
|  | *N* = 28 | |  | *N* = 37 | |  |  |
| **Wake time since preceding nap (min)** | **113.37 (9.22)** | **22.85-209.95** |  | **263.16 (14.95)** | **79.50-420.58** |  | **< 001** |

**Table S3**

Sleep spindle characteristics

|  | **2 - 3 months** | |  | **14 - 17 months** | |
| --- | --- | --- | --- | --- | --- |
|  | ***mean*** | ***SEM*** |  | ***mean*** | ***SEM*** |
| **frequency (Hz)** |  |  |  |  |  |
| frontal | 13.61 | 0.13 |  | 13.65 | 0.13 |
| central | 13.48 | 0.13 |  | 13.74 | 0.12 |
| parietal | 13.17 | 0.09 |  | 13.94 | 0.13 |
| **frequency (Hz) – estimated marginal means** |  |  |  |  |  |
| frontal | 14.20 | 0.14 |  | 13.40 | 0.12 |
| central | 14.10 | 0.14 |  | 13.40 | 0.12 |
| parietal | 13.70 | 0.14 |  | 13.60 | 0.12 |
| **density (# per minute)** |  |  |  |  |  |
| frontal | 3.76 | 0.16 |  | 3.30 | 0.08 |
| central | 3.66 | 0.16 |  | 3.31 | 0.09 |
| parietal | 3.65 | 0.15 |  | 2.64 | 0.10 |
| **amplitude (µV)** |  |  |  |  |  |
| frontal | 27.38 | 1.59 |  | 43.81 | 1.58 |
| central | 32.48 | 1.94 |  | 39.77 | 1.46 |
| parietal | 21.68 | 1.03 |  | 22.65 | 0.66 |

Please note that actual means deviated from the estimated marginal means in the model for spindle frequency. Since we found no discrepancy between actual means and estimated marginal means for any other model, only actual means are reported for the remainder of the manuscript.

**Table S4**

Slow oscillation characteristics

|  | **2 - 3 months** | |  | **14 - 17 months** | |
| --- | --- | --- | --- | --- | --- |
|  | ***mean*** | ***SEM*** |  | ***mean*** | ***SEM*** |
| **density (# per minute)** |  |  |  |  |  |
| frontal | 5.19 | 0.14 |  | 4.72 | 0.11 |
| central | 4.99 | 0.13 |  | 4.93 | 0.10 |
| parietal | 4.70 | 0.13 |  | 4.97 | 0.10 |
| **amplitude (µV)** |  |  |  |  |  |
| frontal | 205.70 | 6.68 |  | 246.88 | 6.71 |
| central | 206.79 | 7.24 |  | 257.14 | 6.70 |
| parietal | 173.84 | 5.63 |  | 237.88 | 6.87 |
| **slope (µV/s)** |  |  |  |  |  |
| frontal | 446.25 | 12.40 |  | 629.08 | 18.33 |
| central | 464.24 | 13.73 |  | 701.88 | 20.87 |
| parietal | 384.76 | 9.78 |  | 663.89 | 22.28 |
| **duration (s)** |  |  |  |  |  |
| frontal | 1.24 | 0.02 |  | 1.12 | 0.01 |
| central | 1.24 | 0.02 |  | 1.12 | 0.01 |
| parietal | 1.23 | 0.02 |  | 1.09 | 0.01 |

**Table S5**

SO-spindle co-occurrence rate

|  | **2 - 3 months** | |  | **14 - 17 months** | |
| --- | --- | --- | --- | --- | --- |
|  | ***mean*** | ***SEM*** |  | ***mean*** | ***SEM*** |
| **observed (percentage of spindles co-occurring with an SO)** |  |  |  |  |  |
| frontal | 10.25 | 0.53 |  | 13.34 | 0.71 |
| central | 10.03 | 0.56 |  | 13.28 | 0.47 |
| parietal | 9.89 | 0.66 |  | 12.32 | 0.47 |
| **chance (percentage of non-REM sleep with SOs)** |  |  |  |  |  |
| frontal | 10.30 | 0.39 |  | 8.74 | 0.24 |
| central | 9.87 | 0.35 |  | 9.13 | 0.23 |
| parietal | 9.23 | 0.36 |  | 8.94 | 0.21 |


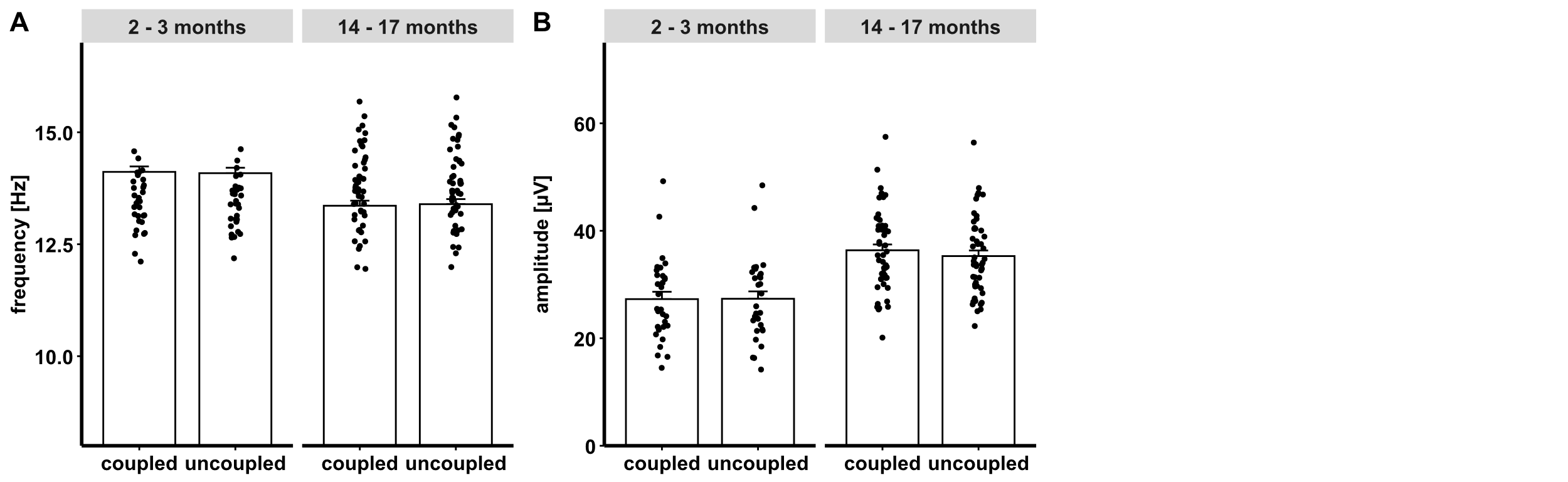


**Figure S1.** Spindle characteristics separately for co-occurring and non-co-occurring events. Please note, estimated marginal means are depicted in A.


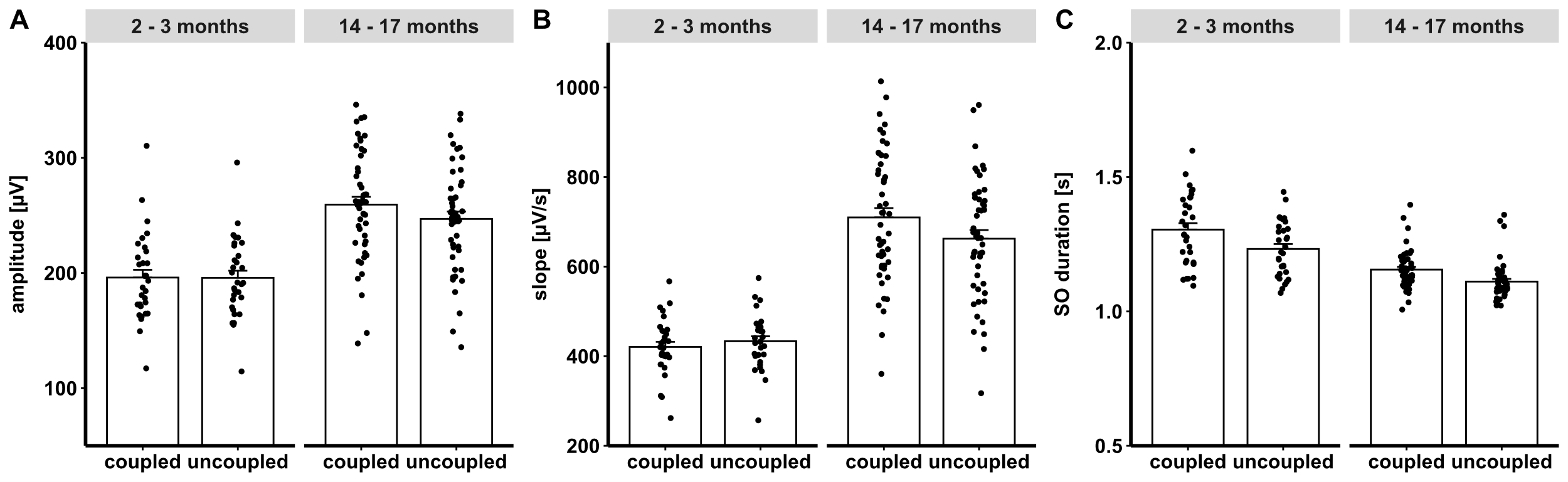


**Figure S2.** Slow oscillation characteristics separately for co-occurring and non-co-occurring events
